# Targeted Gene Insertion for Functional CFTR Restoration in Airway Epithelium

**DOI:** 10.3389/fgeed.2022.847645

**Published:** 2022-03-07

**Authors:** Cristina Barillà, Shingo Suzuki, Andras Rab, Eric J. Sorscher, Brian R. Davis

**Affiliations:** ^1^ Center for Stem Cell and Regenerative Medicine, Brown Foundation Institute of Molecular Medicine, McGovern Medical School, University of Texas Health Science Center at Houston, Houston, TX, United States; ^2^ Department of Pediatrics, Emory University School of Medicine, Atlanta, GA, United States

**Keywords:** cystic fibrosis, CFTR, gene editing, basal cells, gene insertion

## Abstract

Cystic Fibrosis (CF) is caused by a diverse set of mutations distributed across the approximately 250 thousand base pairs of the *CFTR* gene locus, of which at least 382 are disease-causing (CFTR2.org). Although a variety of editing tools are now available for correction of individual mutations, a strong justification can be made for a more universal gene insertion approach, in principle capable of correcting virtually all *CFTR* mutations. Provided that such a methodology is capable of efficiently correcting relevant stem cells of the airway epithelium, this could potentially provide life-long correction for the lung. In this Perspective we highlight several requirements for efficient gene insertion into airway epithelial stem cells. In addition, we focus on specific features of the transgene construct and the endogenous *CFTR* locus that influence whether the inserted gene sequences will give rise to robust and physiologically relevant levels of CFTR function in airway epithelium. Finally, we consider how *in vitro* gene insertion methodologies may be adapted for direct *in vivo* editing.

## Introduction

Cystic Fibrosis (CF) is an inherited recessive disease that results from mutations in the *Cystic fibrosis transmembrane conductance regulator* (*CFTR*) gene (Sharma and Cutting, 2020). In addition to the large number of identified causative *CFTR* mutations, there are over 2000 variants in the *CFTR* gene that may also be responsible for or contribute to CF disease (CFTR2.org; Sharma and Cutting, 2020). As one considers utilizing gene editing tools to correct *CFTR* mutations, one possibility is to develop mutation-specific therapeutic reagents, each capable of correcting at most a small number of mutations. This approach can either be achieved via sequence-specific nucleases (e.g. CRISPR/Cas9, Zinc Finger Nucleases (ZFNs), TALENs) along with corrective donor DNA or with tools that do not require the induction of a double stranded DNA (dsDNA) break (e.g. Base Editors (Levy et al., 2020; Zeng et al., 2020)), Prime Editing (Anzalone et al., 2019). However, there is a strong interest in developing a more universal approach in which a single gene editing therapeutic could be employed to treat and potentially cure nearly all CF patients irrespective of their *CFTR* genotype. It is most likely that the immediate focus for application of such a therapy would be the approximately 7% of individuals with CF who are unable to benefit from modulators due to an insufficient amount of CFTR protein (e.g. due to premature termination codons (PTCs) or splicing mutations). However, once demonstrated to be beneficial in these CF individuals, it is anticipated that such a therapy, potentially a one-time cure, would also be of value to individuals who are responsive to modulators.

This Perspective is primarily focused on the requirements for successful development of such a novel gene therapeutic for CF, using sequence-specific methodologies to target integration of *CFTR* cDNA sequences into the endogenous *CFTR* locus of CF patient-specific airway cells. Directly targeting either a partial or full-length *CFTR* cDNA into the endogenous *CFTR* locus has the potential advantage of correcting or compensating for all *CFTR* mutations downstream of the integration site. To correct for all or nearly all *CFTR* mutations, it likely would be necessary to target integration of the partial *CFTR* cDNA sequences into the most upstream region of *CFTR* sequences, for example from exon 1 thru intron 2. If this can be achieved while retaining the native *CFTR* chromatin structure and regulatory sequences, it also has the possibility of restoring appropriate cell-type specific expression. It is important to note that, within the airway, *CFTR* mRNA is expressed in a cell type-specific manner—being expressed at high level per cell in ionocytes, at low level in secretory and basal cells, and either absent or at very low level in ciliated cells; however, given the relative rarity of ionocytes in airway epithelium, secretory cells appear to contribute the bulk of total *CFTR* mRNA expression (Carraro et al., 2021; Okuda et al., 2021). It is this pattern of regulated expression that one would seek to retain through editing. Although an alternative safe-harbor TI approach for expression of exogenous *CFTR* is possible (Ramalingam et al., 2013), the highly regulated cell type-specific expression of *CFTR* mRNA suggests that directly editing the endogenous *CFTR* locus will likely be necessary to restore appropriate levels of corrected CFTR per cell.

The fore-mentioned basal cells function as stem cells within the airway, being capable of self-renewing cell division as well as giving rise to differentiated progeny including secretory cells, ciliated cells and ionocytes (King et al., 2020). Thus, they are considered to be a preferred target for long-term efficacious *CFTR* gene editing. Genomic editing of the *CFTR* locus in stem cells would ensure that the correction is permanently encoded, while stem cell self-renewal and multi-lineage differentiation would, in principle, ensure long-lasting restoration of normal CFTR function. Based on evaluations of CFTR channel activity in air-liquid interface cultures seeded with mixtures of CF and non-CF airway epithelial cells, the frequency of basal cells requiring gene insertion (for either *ex vivo* editing followed by transplantation or direct *in vivo* editing) to restore CFTR activity to the CF airway is estimated to be on the order of 15–30% (Farmen et al., 2005) or perhaps even less (Lee et al., 2020). In principle, CF patient-specific airway basal cells could be obtained from the airways of individuals with CF (e.g. via brushing), corrected and expanded *ex vivo*, and transplanted back into the airways following appropriate pre-conditioning. One estimate of the total number of basal stem cells required for transplantation of the human conducting airway is of the order of 60 million cells (Hayes et al., 2019). Being an autologous cell-based approach, this therapy would minimize concerns of immune rejection and/or complications of immunosuppressive therapies. An alternative possibility would be to correct CF patient-specific induced pluripotent stem cells (iPSCs), derive airway basal cells (Hawkins et al., 2021), and transplant into the airways. Developing gene insertion strategies for *ex vivo* modification of airway basal cells is considered first in this Perspective. Later we will consider how such *ex vivo* methodologies would need to be adapted for direct *in vivo* editing of the airway.

## *Ex Vivo* Targeted *CFTR* Gene Insertion

### How to Achieve Efficient Targeted Gene Insertion?

Targeted integration (TI), utilizing sequence-specific nucleases, has been shown capable of inserting transgene sequences at specific genomic sites. In theory, TI can be achieved either via homology directed repair (HDR; utilizing flanking homology sequences) or non-homologous end joining (NHEJ)-mediated end capture.

For efficient nuclease-mediated editing utilizing a donor template for TI, there are at least two primary requirements: 1) robust sequence-specific cleavage at the target site; and 2) sufficient delivery of donor DNA template to drive the TI event, rather than the default pathway resulting in NHEJ-induced indels. Thus far, demonstrations of targeted *CFTR* cDNA gene insertion in the endogenous *CFTR* locus of primary airway basal cells have utilized CRISPR/Cas9 or ZFNs to introduce a dsDNA break together with AAV-6-mediated delivery of donor sequences. The *CFTR* sequences targeted for insertion were flanked by homology sequences to facilitate HDR.

Vaidyanathan et al. recently reported CRISPR/Cas9-mediated TI of the *CFTR* cDNA into exon 1 of the *CFTR* locus in airway basal cells. Due to the packaging size constraints of AAV, the *CFTR* cDNA was split into two separate AAV vectors with the *CFTR* cDNA sequences recombining in the target cells. They also incorporated the truncated CD19 (tCD19) coding sequences into one of the AAV-6 vectors. Although the initial TI efficiencies were relatively low (∼5–10%) in the basal cells, likely due to the requirement for co-delivery and recombination, the surface expression of tCD19 allowed selection of TI cells to a 60–80% purity. After differentiation in air-liquid interface (ALI) cultures, restoration of CFTR function was demonstrated (Vaidyanathan et al., 2021).

Our group has recently demonstrated proof-of-principle for this TI approach in minimally expanded CF airway basal cells. TI was first performed in intron 8 of the endogenous *CFTR* gene with a partial *CFTR* cDNA (exons 9–27) preceded by a splice acceptor and terminated with a polyadenylation signal. By generating the sequence-specific dsDNA break in intron (as opposed to exon) sequences, we sought to minimize the potential detrimental effects caused by NHEJ-induced indels at the on-target site. Electroporation-mediated delivery of the ZFN mRNA and AAV-6-mediated delivery of the donor proved to be highly efficient with TI frequencies between 50.0 and 61.8% of *CFTR* alleles in F508del/F508del, F508del/R553X and G542X/R785X basal cells. Importantly, upon differentiation of the targeted basal cells in ALI conditions, intron 8 TI successfully restored mature CFTR protein and channel function at therapeutically-relevant levels (Suzuki et al., 2020). We note that these high rates of TI, also achieved in targeting of intron 7, together with the restoration of CFTR protein and function, were obtained without any selection. We quantitatively assessed, in several single-cell derived clones exhibiting homozygous intron 8 TI, what fraction of *CFTR* mRNA transcripts from targeted alleles were spliced from the endogenous exon 8 to the transgenic exon 9 (thus including transgenic corrective sequences) vs. alternative splicing downstream to the endogenous exon 9 (including only endogenous mutant sequences). Quantitative RT-PCR demonstrated that the majority (58.0—89.9%) of *CFTR* transcripts in these homozygous intron 8 TI clones exhibited the desired splicing into the corrective human codon-optimized exon 9 sequences; however, the remaining transcripts reflected splicing across the corrective exon sequences directly into endogenous exon 9 sequences.

**FIGURE 1:**
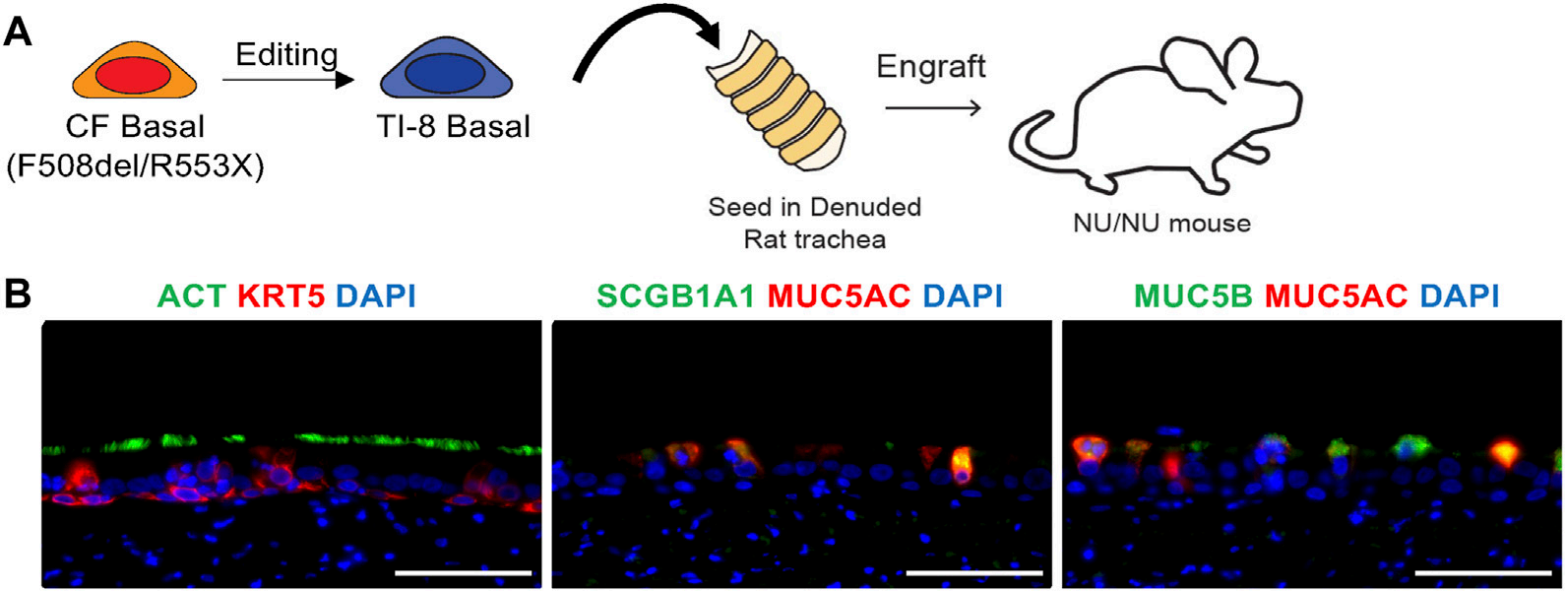
*In vivo* engraftment of edited basal cells (BCs) in tracheal xenografts. **(A)** Schematic of tracheal xenograft. Bulk TI-8 F508del/R553X BCs were seeded in denuded trachea and implanted into the flank of a Nu/Nu mouse. **(B)** Identification of basal (KRT5), secretory (SCGB1A1, MUC5AC, MUC5B), and multiciliated (ACT) cells using immunostaining. DAPI staining indicates DNA (Scale bar = 50 μm).

Given this efficient gene insertion *ex vivo* in airway basal cells, we wished to demonstrate the competence of edited cells to establish a well-differentiated airway epithelium *in vivo*. Thus, a bulk population of TI-8 edited F508del/R553X basal cells were seeded into denuded rat tracheas and implanted in nu/nu mice (Filali et al., 2002; Hawkins et al., 2021) (Figure 1A). We observed the development of airway epithelium in seeded tracheas. Immunostaining confirmed multipotent differentiation with the clear presence of basal, secretory, and ciliated cells (Figure 1B).

**FIGURE 2:**
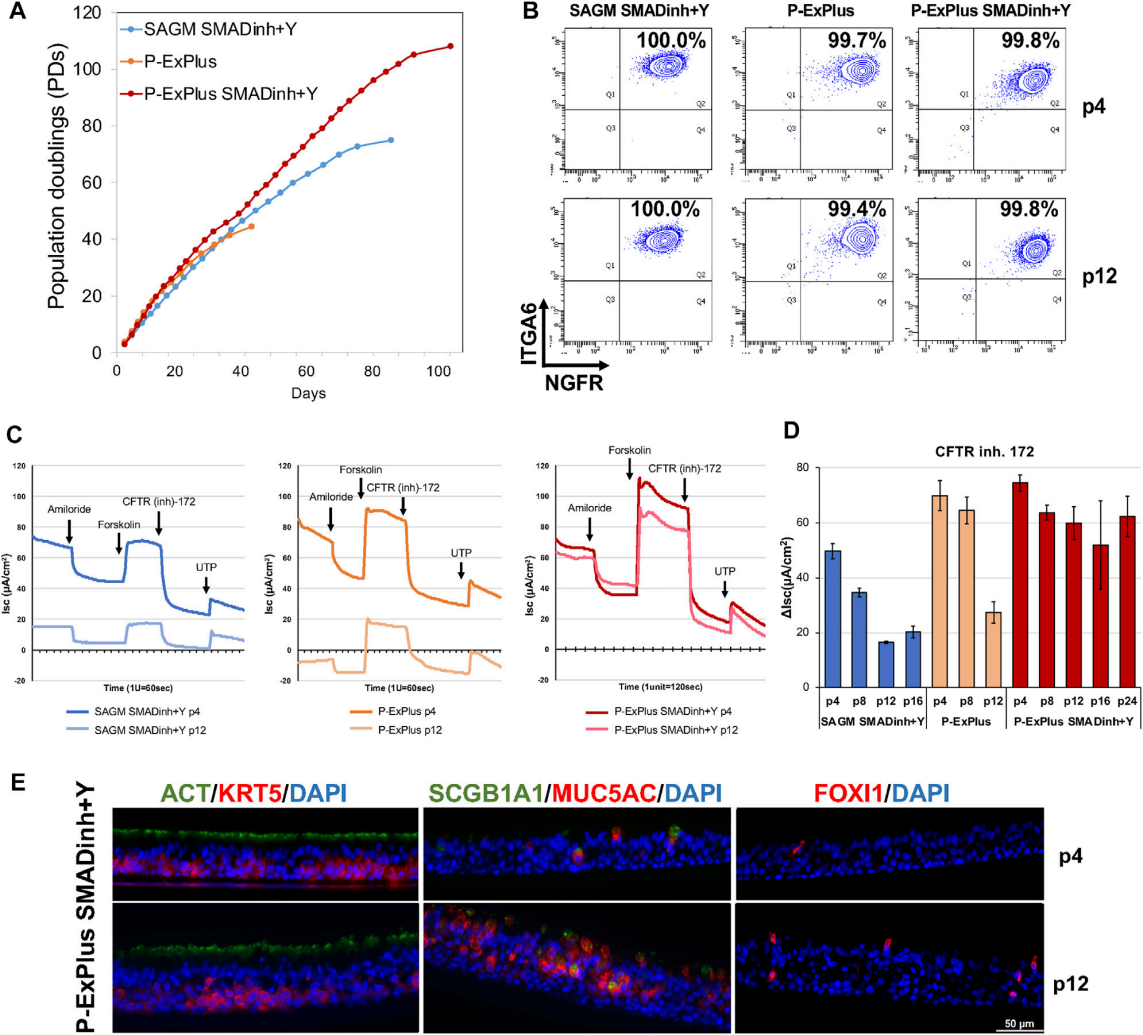
Long-term expansion of BCs with retention of CFTR function. **(A)** Growth curve of BCs cultured in Small Airway Growth Medium with SMAD inhibitors and ROCK inhibitor (SAGM/SMADinh + Y), and Pneumacult-Ex Plus with or without SMADinh and Y (P-ExPlus/SMADinh + Y or P-ExPlus). **(B)** Flow cytometry analysis of cells at passage 4 (p4) and p12 in **(A)**. Cells are immunolabeled for airway basal cell markers, ITGA6 and NGFR. **(C)** Individual electrophysiological tracings for ALIs at p4 and p12 in the indicated media; times of acute treatments with amiloride, forskolin, and CFTR inhibitor-172 are indicated. **(D)** Ussing chamber analysis of ALIs derived from basal cells over the culture in **(A)**. The graph shows the current change in response to CFTR inhibitor-172. Shown are mean ± SD for 3-4 transwells per condition. **(E)** Transverse-section of ALIs derived from BCs expanded in P-ExPlus/SMADinh + Y at p4 and p12. Immunofluorescence staining for ionocytes (FOXI1), ciliated (ACT), secretory (SCGB1A1, MUC5AC) and basal (KRT5) cells. Scale bar = 50 μm.

We seek to develop culture methods enabling feeder-free expansion of edited airway basal cells to obtain a sufficient cell number for transplantation while retaining CFTR function. Such methods could be applied to bulk edited cells (i.e. mixture of corrected and uncorrected cells) or to corrected single-cell derived clones. We previously documented (Suzuki et al., 2020) that basal cells expanded in SAGM medium supplemented with SMAD inhibitors and ROCK inhibitor (SAGM/SMADinh + Y) or in Pneumacult-ExPlus (P-ExPlus) were capable of significant expansion as basal cells (Figures 2A,B). However, when cultured in either medium for greater than 8 passages and then differentiated in ALI culture, the level of CFTR-dependent current decreased significantly (Figure 2C). We note that achieving the fore-mentioned 60 million cells corresponds to approximately 26 population doublings (approximately 8 passages) starting with only one basal stem cell; in our experience, airway brushing of CF airways yields a far greater number of starting cells (data not shown). We have since found that supplementing P-ExPlus with SMAD inhibitors and ROCK inhibitor (P-ExPlus/SMADinh + Y), enables even greater expansion (Figure 2A), retention of long-term basal cell markers (e.g. ITGA6, NGFR) (Figure 2B), as well as multipotential differentiation, with a more stable and thicker epithelium consisting of basal, ciliated, secretory cells and ionocytes (Figure 2D). In addition, we now have superior maintenance of CFTR channel activity (Figure 2C). This last finding is highly significant in that we are unaware of any methodology, with or without feeders, able to extensively expand human airway basal cells without significant loss of CFTR activity. Future studies will include basal cells derived from multiple donors to confirm this finding.

Whereas HDR-mediated TI requires cells to be in cell cycle, NHEJ-mediated end capture of AAV transduced sequences has also been demonstrated in non-dividing cells (Suzuki et al., 2016). Typically, NHEJ-mediated TI integrates donor sequences equally in forward or reverse directions. To maximize the desired directionality of integrated sequences via CRISPR/Cas9, gRNA and protospacer adjacent motif (PAM) recognition sequences are directionally incorporated in the donor to favor the correct orientation (known as Homology Independent TI; HITI (Suzuki et al., 2016)). A rather similar directional approach utilizing obligate heterodimer ZFNs has been developed (known as ObLiGaRe (Maresca et al., 2013; Nami et al., 2018)).

Although sequence-specific nucleases are able, in many cases, to achieve highly efficient TI (either via HDR or NHEJ end capture), such conditions frequently result in indels (in some cases significantly sized deletions) at the target site of alleles not achieving TI. In addition, off-target indels may result in unwanted and unanticipated adverse consequences. Thus, there is significant interest in developing gene insertion methodologies that do not require a dsDNA break for sequence-specific targeting, such as transposon-encoded CRISPR/Cas systems (Klompe et al., 2019), twin prime editing (Anzalone et al., 2021), and fusion of CRISPR/Cas9 nickase to both a reverse transcriptase and serine integrase (Ioannidi et al., 2021). Alternatively, there are ongoing efforts to confer sequence-specificity upon retroviral or lentiviral vectors that typically do not exhibit sequence specific integration (Yoder et al., 2021).

There are several considerations relevant to determining which *CFTR* intron is optimal for TI. One consideration would be to maximize the number of CF patients (or *CFTR* mutations) that could, in principle, benefit from site-specific TI. For example, intron 8 TI would provide correction for 89.1% of the CF-causing *CFTR* alleles listed in the CFTR2 database including F508del, common PTCs (e.g. G542X, R785X, and W1282X), and splicing variants (e.g. 3,849 + 10 kb C > T) (Suzuki et al., 2020), whereas exon 1 (Vaidyanathan et al., 2021) or intron 1 targeting would provide correction for nearly all *CFTR* mutations. A second consideration is the size of the requisite *CFTR* partial cDNA and whether this can be appropriately delivered with a chosen vector; thus, the use of two AAV vectors for exon 1 TI (Vaidyanathan et al., 2021). A third consideration involves the relative strength(s) of the transgene splice acceptor vs. the native splice acceptor in the immediate downstream *CFTR* exon. A fourth aspect comprises the need to achieve transgene integration without disrupting activity of critical cis regulatory elements (CREs) and the chromatin architecture of the *CFTR*. We discuss the latter two issues in the following section.

### How to Achieve Appropriately Regulated Expression of Corrected CFTR?

By directly integrating the partial or full-length *CFTR* cDNA transgene into the *CFTR* locus, the objective would be to obtain physiologically- and therapeutically-relevant levels of corrected CFTR. In so doing, there are several considerations:

*1*) *How to maximize incorporation of corrective transgene sequences in the mRNA and protein?* Transgenic mouse studies showed that maintaining some intron/exon structure gave rise to higher expression of the transgene. As previously mentioned, it is important to consider the relative strength(s) of the transgene splice acceptor vs. the native splice acceptor in the immediate downstream *CFTR* exon. If the targeted intron were immediately upstream of a strong splice acceptor sequence, it is possible that a significant amount of splicing could jump across the corrective partial *CFTR* cDNA. A failure to capture only corrective cDNA sequences in *CFTR* mRNA transcripts from successfully targeted alleles, as observed in our published study (Suzuki et al., 2020), would potentially result in reduced levels of CFTR protein and activity. If splicing across the transgene were to be an issue, this could potentially be resolved by strengthening the splice acceptor sequence, varying the poly-adenylation sequence to maximize termination of transgene transcription, or perhaps changing the insertion site within the intron.
*2*) *Whether to utilize native vs. codon-optimized transgene?* In principle, codon optimization may enable higher levels of transgene expression (Marquez Loza et al., 2021). We note that the fore-mentioned example of locus-specific TI in basal cells employed codon-optimized transgenes (Suzuki et al., 2020). However, it is possible that use of non-native amino acid-encoding transgene sequences (i.e. human codon-optimized) may influence the efficiency of generating the appropriately folded CFTR protein necessary for functional activity (Kim et al., 2015). Furthermore, the level of CFTR function resulting from lentiviral delivery of various codon-optimized *CFTR* transgenes depended on the algorithm used for the optimization (Marquez Loza et al., 2021). This perhaps highlights, for TI applications, the importance of assaying the consequences of codon optimization when the *CFTR* transgene is integrated into the *CFTR* locus with its expression directed by the endogenous promoter.
*3*) *How to maintain CFTR chromatin architecture?* The regulation of *CFTR* expression is governed by a three-dimensional (3D) chromatin architecture and by specific regulatory DNA sequences (e.g. CREs), the function of which can vary between various CFTR-expressing cells/tissues. To achieve physiologically-regulated *CFTR* expression, one would wish to minimally disrupt this 3D architecture and the role of specific regulatory elements. This can be considered as an interplay between chromatin and transgene—with the inserted transgene potentially influencing chromatin structure and the chromatin architecture governing transgene expression. For example, our intron 8 TI had minimal impact on the native *CFTR* locus open chromatin profile (Suzuki et al., 2020).

## *In Vivo* Targeted *CFTR* Gene Insertion

To apply the fore-mentioned gene insertion approaches for direct *in vivo* modification of airway basal cells, it will be important to deal with delivery and cell cycle status. We briefly summarize these issues below:

### How to Achieve Delivery of TI Reagents to Airway Basal Cells?

One of the primary challenges to efficient TI of airway basal cells *in vivo* will be the delivery of the editing reagents. In principle, luminal delivery to the airway epithelium is possible via viral (e.g. AAVs) or non-viral (e.g. lipid nanoparticles) (Robinson et al., 2018) methodologies provided that the CF mucus barrier can be penetrated or transiently removed. Given the tight junctions that characterize the pseudostratified airway epithelium, it remains challenging to deliver editing reagents to the basolaterally located basal cells. Treatment of human airway epithelium by transiently disrupting tight junctions or inhibiting proteasomal processing facilitates AAV transduction (Coyne et al., 2000; Duan et al., 2000; Yan et al., 2004; Ellis et al., 2013). Systemic delivery of editing reagents via the bloodstream is another possibility to access the airway epithelium, including the basal cells. There have recently been significant advances in the development of various non-viral vectors, including nanoparticles, for *in vivo* targeting of specific organs via systemic administration. For example, it has been shown that it is possible, via systemic delivery, to successfully target delivery of Cas9 to the lung (Cheng et al., 2020). Targeting delivery of editing reagents specifically to basal cells (e.g. via recognition of surface-expressed NGFR) could perhaps maximize the efficiency of editing in these cells.

### How Will Cycling Status of Basal Cells Dictate TI Strategies?

Only a low frequency of airway basal cells is generally cycling at any given time in the non-CF human lung. However, the frequency of cycling airway basal cells in CF lungs is reportedly higher, which is likely due to ongoing inflammation and repair of damage (Voynow et al., 2005). In contrast to these findings, however, Carraro et al. recently determined via scRNA-seq that the frequency of basal cells in CF airways that are cycling was less than in control non-CF airways (Carraro et al., 2021). To facilitate efficient gene insertion into the significant fraction of the CF airway basal cells that are non-dividing at any given point in time (likely to be >75%) (Voynow et al., 2005), we must also consider alternative approaches to HR-mediated TI. Since the NHEJ repair mechanism, as well as other targeting approaches, are also active in non-cycling cells, such methods may be required, e.g. HITI (Suzuki et al., 2016), prime editing (Anzalone et al., 2019), twin prime editing (Anzalone et al., 2021), transposon-encoded CRISPR/Cas system (Klompe et al., 2019), and fusion of CRISPR/Cas9 nickase to both a reverse transcriptase and serine integrase (Ioannidi et al., 2021).

## Discussion

Recent developments in various methodologies, including gene editing, *ex vivo* expansion of airway basal cells, and *in vivo* delivery of editing reagents to the lung, offer hope that effective, universal, targeted gene insertion-based therapies may eventually be developed for the CF airway. Although our focus in this Perspective has been on correction of airway basal cells for long-term (potentially life-long) benefit, it may be important to additionally target the correction of differentiated cell types (including secretory cells and ionocytes) since it is unknown how long it will take for a corrected airway epithelium to be derived from the corrected basal cells. This Perspective has been focused on correction of the CF conducting airway, particularly the pseudostratified epithelium of the large airway. However, it is very possible that other regions of the lung (e.g. small airways, submucosal glands in the trachea and bronchi) may also need to be corrected for maximum clinical benefit. Finally, systemic delivery of editing reagents would potentially extend *in vivo* editing of *CFTR* mutations to affected organs other than the lung (e.g. pancreas, intestine).

## Methods

### Culture, Characterization, and *In Vitro* Differentiation of Airway Basal Cells

DD023J (non-CF airway epithelial cells) and KK002C (CF airway epithelial cells: F508del/R553X *CFTR*), obtained from explanted lungs, were provided by the Tissue Procurement and Cell Culture core facility at the University of North Carolina, NC, United States. The details of culture, characterization and *in vitro* differentiation of airway basal cells were previously described (Suzuki et al., 2020) In addition to the two culture media previously described (Suzuki et al., 2020) Pneumacult™-Ex Plus (STEMCELL technologies, Vancouver, Canada) medium and SAGM™ medium (Lonza, Basel, Switzerland) supplemented with dual SMAD inhibitors, we tested dual SMAD inhibition medium consisting of Pneumacult™-Ex Plus supplemented with 10 µM RhoA kinase (ROCK) inhibitor Y27362 (Reagents Direct, Encinitas), 1 µM A-8301 (R&D Systems, Minneapolis, MN), and 1 µM DMH-1 (R&D Systems). In all three culture conditions, airway basal cells were cultured on pre-coated plates with laminin-enriched 804G cell-conditioned medium and split at a 1:10 ratio upon their confluency at 50—70%. DD023J cells were used to test their capabilities of long-term expansion in each medium and characterized with known basal cell surface markers CD49f and NGFR using flow cytometric analysis. Cells at several passages from each medium were differentiated *in vitro* under identical air liquid interface (ALI) condition.

### Tracheal Xenograft Assay

The F508del/R553X *CFTR* KK002C cells, subjected to intron 8 targeted integration (Suzuki et al., 2020), were expanded in Pneumacult™-Ex Plus medium and assayed in the tracheal xenograft model. The methodology was as reported (Filali et al., 2002) and as recently utilized by our group (Hawkins et al., 2021).

### Ussing Chamber Analysis

Epithelial monolayers were established under ALI conditions in transwells from non-CF (DD023J) airway basal cells originally cultured in the fore-mentioned media for various number of passages. Ussing chamber experiments were performed as previously described (Suzuki et al., 2020); the acquired data, analyzed for 3-4 transwells per condition, are expressed as mean ± SD.

## Data Availability

The original contributions presented in the study are included in the article, further inquiries can be directed to the corresponding author.
